# Development of a Three-Component Nutrient Density Calculator (NDC) for Mixed Dishes to Guide Innovation and Reformulation of Recipes

**DOI:** 10.3390/nu18142282

**Published:** 2026-07-12

**Authors:** Matthieu Maillot, Veruska Calabretta, Jackie Schulz, Angela Bonnema, Adam Drewnowski

**Affiliations:** 1 MS-Nutrition, 13005 Marseille, France; matthieu.maillot@ms-nutrition.com; 2 Griffith Foods, Pioltello, 20096 Milan, Italy; 3 Griffith Foods, Alsip, IL 60803, USA; jschulz@griffithfoods.com (J.S.);; 4 Center for Public Health Nutrition, University of Washington, Box 353410, Seattle, WA 98195, USA

**Keywords:** Nutrient Density Calculator, serving size, mixed dishes, recipes, ingredients, Nutrient Rich Food (NRF) index

## Abstract

Background: Capturing the healthfulness of mixed dishes per serving can be a challenge. Objectives: To develop a Nutrient Density Calculator (NDC) for mixed dishes to guide recipe innovation and reformulation. Methods: Recipe files and nutrient composition data for mixed dishes came from the US Department of Agriculture Food and Nutrient Database for Dietary Studies (FNDDS 2021–2023). Included were mixed dishes with meat, poultry, seafood, grains, vegetables or legumes as their main ingredient, Asian and Mexican dishes, pizza, sandwiches, burgers, and nuts and seeds (*n* = 1570). Observed servings as consumed came from the National Health and Nutrition Examination Survey data (NHANES 2017–2020/2021 –2023). The NDC version 643 had three components: NR6 based on six nutrients to encourage (protein, fiber, calcium, iron, potassium, vitamin D); MP4 based on ingredients (fruits and vegetables, whole grains, pulses, nuts and seeds); and LIM based on three nutrients to limit (saturated fat, added or total sugar, sodium). An abbreviated version, NDC343, used NR3 (protein, fiber, calcium). Alternative NDC models tested different component weights. Nutrient density was calculated per serving. Results: Both NDC643 and NDC343 gave higher scores to vegetables, pulses, and nuts and lower scores to meat-based dishes. Equal weighting of the three components produced NDC scores that were correlated with Nutri-Score (r = −0.64 for NDC343 and −0.70 for NDC643) and Health Star Rating values (r = −0.74 for NDC343 and −0.75 for NDC643). Correlations with energy density were −0.25 for NDC343 and −0.33 for NDC643. The NDC643 model was sensitive to recipe improvements for a variety of mixed dishes—more so than the Nutri-Score or the Health Star Rating. Conclusion: The three-component NDC model, combining both nutrients and ingredients, successfully captured nutrient density of mixed dishes and was sensitive to recipe improvements.

## 1. Introduction

Calculating the nutrient density of composite mixed dishes presents a number of challenges. First, existing nutrient profiling (NP) systems, including Nutri-Score and the Health Star Rating, were originally designed to evaluate the healthfulness of processed packaged foods [1,2,3]. Intended to prevent overconsumption and overweight, such NP models penalize calories, fat, sugar, and salt, generally regarded as nutrients to limit [1,2,3]. There is less emphasis on positive nutrients to encourage, such as protein, fiber, vitamins and minerals [1]. Second, desirable ingredients such as whole grains are not always taken into account [4]. Third, calculations are performed per 100 g and different serving sizes are not considered [1,2,3].

While NP models can be consumer facing, they can also guide product reformulation by the food industry [5]. Efforts are under way to reduce the saturated fat, added sugar and salt contents of packaged processed foods. Prepared meals, some offered by the restaurant industry, are being reformulated as well. Based on industry reports, fast food chains are reformulating menus in 2025–2026 to enhance flavor and improve nutrient density. Major nutrition-relevant upgrades have focused on buns, sauces, and preparation methods. Those recipe changes might not be captured by existing NP methods [4].

Nutrient density calculations depend on the availability of reliable nutrient composition data [6]. Although nutrient composition of mixed dishes can be assayed in direct laboratory analysis [7,8,9,10], most nutrient composition databases use recipes, summing up the energy and nutrient contents of recipe ingredients, adjusted for preparation, yield and loss [11,12,13]. The US Department of Agriculture Food and Nutrient Database for Dietary Studies (FNDDS) [6] does include a standardized recipe file with the needed ingredient information. Those calculated nutrient values are approximations that may not be equivalent to the lab-assayed nutrient content [7]. When ingredients are combined and cooked, nutrients can degrade, transform, or interact in ways that are not captured by summing ingredient nutrient values.

The existing NP methods typically use standard weight (100 g) or calories as the basis of calculation. Both Nutri-Score and Health Star Rating (HSR) use 100 g as the reference amount, whereas the Nutrient Rich Food (NRF) index uses 100 kcal [14]. That approach does not help answer the question of which provides more nutrients: a burger, a sandwich, or a slice of pizza?

Mixed dishes available in restaurants, including quick service restaurants, are generally sold in pre-determined portion sizes [15]. The hospitality industry and restaurants tend to standardize both recipes and portion sizes. However, different suppliers or restaurants may produce very different versions of the same mixed dish [15]. A serving of pasta, pizza or stir-fry can vary depending on how the food was made, where it was bought, and how it was served. Even if the recipes are standardized, serving sizes are not. The amounts consumed may not correspond to the US Food and Drug Administration (FDA) Reference Amounts Customarily Consumed (RACCs) [16].

The present goal was to develop a Nutrient Density Calculator for mixed dishes that would combine both nutrients and desirable ingredients. Significantly, the basis of calculation would be serving size, as opposed to 100 g or 100 kcal [14]. To simplify matters, we opted for a point score that was closer to the letter grades for Nutri-Score [2] or the five stars for the Health Star Rating or HSR [17]. One requirement was that the Nutrient Density Calculator should be sensitive to any changes or improvements in recipe ingredients. The intent was to guide the hospitality, prepared meals, and packaged foods industries toward innovation and potential reformulation of product recipes [1,5].

## 2. Materials and Methods

### 2.1. Food and Nutrient Database for Dietary Studies (FNDDS 2021–2023)

The US Department of Agriculture FNDDS is the nutrient composition database used in the What We Eat in America (WWEIA) studies [6], the dietary component of the National Health and Nutrition Examination Survey (NHANES). Data per 100 g, edible portion, are available for energy, protein, carbohydrate, total sugars, total lipids, saturated fats, fiber, vitamins (A, B1, B2, B3, B5, B9, B12, C, E) and minerals (iron, calcium, potassium, zinc, iodine, sodium, magnesium).

Mixed dishes listed in the FNDDS 2021–2023 were mixed dishes with meat, poultry, seafood, grains or beans as the main ingredient, Asian or Mexican dishes, pizza, sandwiches, and burgers (*n* = 1320). We also included mixed foods (*n* = 172) from other categories, notably beans, peas and legumes, coleslaw, mashed potatoes, and French fries along with nuts and seeds (*n* = 78). The final analytical sample was 1570 foods aggregated into categories using 4-digit WWEIA codes [6] (Table 1).

### 2.2. Food Pyramid Equivalents Database (FPED) 2017–2020

Mixed dishes selected were matched to FPED 2017–2020 database [18] which values for whole grains and added sugars were taken from. In FPED, cooked grains such as cooked rice, pasta, and hot breakfast cereals are converted to uncooked forms, and one ounce equivalent of grains is defined as 28.3 g. The amount of whole grains (in g per 100 g) was calculated using 28.3 g as 1 oz equivalent for all foods containing whole grains except for sandwiches where 16 g equivalent was used. Added sugar content (provided in teaspoon eq. per 100 g) was converted to g/100 g using a conversion factor of 4.2.

### 2.3. FNDDS 2021–2023 Recipe File

The FNDDS recipe file provides a detailed list of ingredients (in grams) for 5431 FNDDS food codes [6]. Some recipes are expressed for 100 g of the final recipe but in other cases the total weight of the recipe can be higher or lower than 100 g. After matching the recipe file to our sample of mixed dishes, ingredient lists were extracted and each ingredient was manually assigned to fruit, vegetable (excluding potatoes), pulse, or nut and seed. Estimated weights of fruits, vegetables, pulses and nuts and seeds in g/100 g were based on the weight (in grams) of each ingredient and the total weight of the recipe. Recipes were not available for 150 foods. For those foods, estimates were based on similar items in FNDDS where gram amounts were available.

### 2.4. Observed Servings and FDA Reference Amounts Customarily Consumed (RACCs)

Dietary intake data on portion sizes came from the NHANES cycles 2017–2020 and 2021–2023 for US adults (*n* = 10,925), aged >19 years and with two 24 h dietary recalls [19]. Observed portion sizes for foods as consumed were calculated across all eating occasions for that food category. The distribution of portion sizes was expressed as mean, standard deviation, median, 10th percentile and 90th percentile. All analyses were survey weighted to be representative of the consumption of the US population. The observed values were compared to the Food and Drug Administration (FDA) Reference Amounts Customarily Consumed or RACCs [15]. RACC values are the mandated serving sizes in the US. Those are featured on the back-of-pack Nutrition Facts Panel for packaged foods.

### 2.5. Nutrient Density Calculator

The three NDC sub-scores or components were based on nutrients to encourage, ingredients to encourage and nutrients to limit. The inclusion of ingredients echoed earlier published hybrid NRF scores that included both nutrients and ingredients [16]. However, there were also substantial differences between the new NDC and the NRF. Whereas the NRF algorithm used sums of percent daily values, the NDC used means. In that respect, the NDC was closer to the French two-component SAINLIM system [20,21], where SAIN (score of nutritional adequacy of individual foods) is based on nutrients to encourage (i.e., proteins, fiber, vitamin C, iron, calcium, vitamin D) and LIM (score of nutrients to be limited) is based on nutrients to limit (i.e., free sugars, sodium and saturated fats). Second, calculations were based on portion sizes as opposed to 100 g or 100 kcal as the reference amount. Third, the number of qualifying nutrients was variable, also similar to SAIN/LIM.

#### 2.5.1. Nutrient Standards

Nutrient standards used to calculate percent daily values came from the FDA [22] and are summarized in Table 2. The FDA standards, expressed for a daily diet of 2000 kcal, are not age- or sex-specific and represent recommended values or the upper limit for individuals aged >4 years.

For ingredients, reference amounts in grams came from the Dietary Guidelines for Americans (DGA) [22] and the Healthy US-Style Dietary Pattern (2000 kcal). The DGA 2020 recommendation for cooked pulses was 1.5 cups of cooked pulses (beans, peas, lentils) per week for a 2000-calorie diet, which corresponds to 30–40 g/day [23]. However, the DGA advisory committee [24] suggested a higher value of 2.5 cups/week, equivalent to 50–66.6 g/day. We used the higher end value of 65 g/day as the standard. For nuts and seeds, the recommended daily intake was about 1 oz. per day (30 g). The same value of 30 g/day is recommended in France, Australia, and the Nordic countries [25,26]. We used 30 g as the standard. The DGA 2020 recommendation for whole grains was 3 oz/day or 48 g/day [23]. We used 50 g/day. The DGA 2020 recommendations were 2.5 cups/day of vegetables and 2 cups/day of fruits [23]. Actual consumption is much lower. For the present purpose, 1 cup eq. of vegetables (equal to 85 g) was the standard used. In other words, to achieve the maximum possible MP4 component (i.e., 100%), one serving of a mixed dish would need to contain 85 g of vegetables, 65 g of pulses, 30 g of nuts and seeds, or 50 g of whole grains.

#### 2.5.2. Three NDC Components and the Overall NDC Score

The NDC was based on 3 separate components. The positive Nutrient Rich component (NRx) estimated mean percentage daily values (DVs) for 3 to 6 nutrients in one serving of food. The NR3 component had 3 nutrients to encourage: protein, fiber and calcium. The NR6 component had 6 nutrients to encourage: protein, fiber, calcium, iron, potassium, and vitamin D. The calculations were as follows:

(1) $${NR}_{x}=\frac{1}{x}\sum_{i=1}^{i=x}Minimum\left(\frac{{Nutrient}_{i}}{{DV}_{i}}\times 100;100\right)$$

which ranges from 0 to 100%.

The positive MyPyramid component (MP4) estimated the mean percentage adequation to reference values (RVs) for 4 ingredient groups in one serving of food. The ingredients to encourage were fruits and vegetables, whole grains, pulses, nuts and seeds.

(2) $${MP}_{4}=\frac{1}{4}\sum_{i=1}^{i=4}Minimum\;\left(\frac{{Ingredient}_{i}}{{RV}_{i}}\times 100;100\right)$$

which ranges from 0 to 100%.

The negative limited nutrients component (LIM3) estimated the mean percentage adequation to maximal recommended values (MRVs) for 3 limiting nutrients in one serving of food. The 3 nutrients to limit were saturated fat, added or total sugars, and sodium.

(3) $${LIM}_{3}=\frac{1}{3}\sum_{i=1}^{i=3}Minimum\;\left(\frac{{Limiting\;Nutrient}_{i}}{{MRV}_{i}}\times 100;100\right)$$

which ranges from 0 to 100%.

The Nutrient Density Calculator (NDCx:y:z) final score was the sum (assuming an equivalent weight) of the qualifying nutrient (NRx) component and the ingredient-based component (MPy) minus a limiting nutrient component (LIMz), as follows:

(4) $${NDC}_{x:y:z}={NR}_{x}+{MP}_{y}-{LIM}_{z}$$

which ranges from −100 to 200%.

In the calculations above,

- *Nutrient_i_* and *ingredient_i_* are the amounts of desirable nutrients or ingredients per serving. *Limiting nutrient_i_* is the amount of limiting nutrients per serving. The LIM component can include total or added sugars depending on availability of data.
- *Serving i* is the estimated mean serving size as consumed by adults in the NHANES 2017–2019 and 2021–2023 data
- *DVi* are the daily values as specified in the FDA Nutrition Facts Panel (Table 2). *RVi* are reference values corresponding to the amount of ingredient per serving (see Table 2). *MRVi* are maximal recommended values as listed in the FDA Nutrition Facts Panel.
- Percent daily values per serving were capped at 100% following NRF standard practice. The capping is indicated by the minimum function in formulas (1)–(3).

#### 2.5.3. Weighting of Sub-Score Components

Three alternative weighting schemes were tested (see Table 3) to give differential weights to positive nutrients (NRn) and ingredients (MP4) and to nutrients to limit (LIM).

The default NDC score (1,1,1) assigned equivalent weights to each of the three components. As a result, the final score favored the NRn and MP4 components. The (2,1,1) version weighted the NRn component even more. By contrast, the (1,1,2) version gave more weight to the negative LIM3 component. The (1,2,1) version gave more weight to the MP4 component. The differential weights did have an impact on the final NDC score. For example, mixed dishes that contained animal versus plant protein were the most affected. For example, plant-based mixed dishes typically contain more fiber and lower amounts of saturated fats and sodium but also less protein. Conversely, animal-based foods contain more protein and some key nutrients but also more sodium and saturated fat.

#### 2.5.4. Nutrient Density Calculator as a 10-Point Score

The NDC is a continuous score ranging from negative to positive values. However, we opted to use a point system to standardize and make easier the interpretation of the different NDC scores and that would be more comparable to Nutri-Score (from letter A to E, equivalent to a 5 points score) or the Health Star Rating (from half star to 5 stars equivalent to a 10-point score). The goal was to facilitate product comparisons across different scoring systems. Cut points for NDC were identified using distribution deciles for a large sample of mixed dishes eaten in the US. Then, threshold values were derived by rounding the decile values. Depending on the NDC values compared to the threshold, each food scored 1 to 10 points. One point indicated the worst nutritional quality rating, while 10 points meant the highest nutritional value.

### 2.6. Comparisons with Existing Nutrient Profiling Metrics

Nutri-Score and the Health Star Rating (HSR) are front-of-pack nutrition labeling systems in current use. Both systems balance nutrients to limit (energy, saturated fat, total sugar, sodium) against two nutrients to encourage: protein and fiber. The positive component also includes product contents of fruits, vegetables, pulses, nuts and seeds. All calculations are per 100 g. Point scores increase with negative nutrients minus the positive component. The Nutri-Score algorithm [2] then calculates total points for a given food item and converts them into a five-color letter grade (from A to E). The HSR follows a similar system but the final score is communicated by a number of stars—from 0.5 to 5.0. The HSR score makes special allowances for dairy foods. All foods in this study were classified as “Solid foods” by Nutri-Score, except nuts and seeds which were assigned to the “Fats, oils, seeds and nuts” group algorithm. For the HSR, mixed foods were assigned to the “2-Foods” category.

### 2.7. Statistical Analyses

Statistical analyses focused on evaluating the internal structure and robustness of the scoring system. Spearman correlation coefficients were calculated between the different score versions, between individual nutrient/ingredient ratios and their corresponding sub-scores, between sub-scores and the overall scores, and across sub-scores themselves. Finally, the scores were compared with the Nutri-Score, the Health Star Rating, the energy density (kcal/100 g) and the energy per serving, using Spearman correlations. The RACC values were compared to the estimated mean portion size consumed in NHANES, using 95% confidence limits to consider a significant difference. Then, the different versions of NDC were centered by subtracting the means and standardized by dividing by the standard deviation. Food categories were ranked according to the means of standardized NDCs. Evolution of the ranking was manually analyzed to better understand the impact of the weighting. Finally, the NDC 10-grade score was used to compare food rankings using the different versions of the NDC643. The percentage of foods assigned grades from 0 to 10 was estimated for each category to assess the NDC’s sensitivity to discriminate between foods within the same category. A food category with few distinct NDC grades would indicate that the score has limited discriminatory power. The weighted Kappa coefficient was also calculated between each pair of 10-point scores derived from NDC643(1,1,1), NDC643(2,1,1), and NDC643(1,1,2). All statistical analyses were realized with SAS software 9.4 (SAS Institute Inc., Cary, NC, USA), considering *p*-values of Spearman correlation lower than 5% as significant.

### 2.8. Sensitivity of the NDC in Food Product Reformulation

The version (1,1,1), (1,1,2), (2,1,1) and (1,2,1) of the NDC643 10-grade score was applied to the 40 food products (e.g., rice dishes, fries, meatballs, chicken, burgers, sandwiches) provided by Griffith Foods. Among these products, some of them were a reformulated recipe of an original one (*n* = 6). Five original recipes have one revised version while one original recipe has 2 revised versions. The nutritional reformulation (percentage increase/decrease in nutrients per serving) used is explained in Supplemental Table S1. The reformulation of flavored rice, Italian meatballs, and flavored peanuts focused on sodium reduction. Sodium reduction was achieved through the use of various reformulation techniques such as the use of potassium chloride along with flavor balancing. Reformulation of Italian meatballs reduced sodium and sugar content and increased total fiber. The chickpea and lentil dish was reformulated by replacing most of the coconut milk with low fat milk. The goal was to determine whether the NDC for mixed foods was sensitive to recipe reformulation through reduction in nutrients to limit or the addition of desirable ingredients.

## 3. Results

### 3.1. Observed Serving Sizes vs. Reference Amounts Customarily Consumed (RACCs)

Consumption data for each food category came from NHANES 2021–2023 [19]. Shown in Table 4 are means, SEM, upper and lower confidence estimates, medians and 10th and 90th percentiles. RACC values for the same categories are also shown. Mean serving sizes for mixed dishes ranged from 199 g for Mexican dishes to 255 g for meat-based dishes. One exception was pasta-based dishes with mean servings of 323 g. Mean servings were 178 g for burgers. Sandwiches ranged from 146 g (vegetable-based sandwich) to 216 g (meat-based sandwich). For the single dishes, the serving size ranged from 95 g (French fries) to 190 g (potato-based dishes). For nuts and seeds the estimated mean serving size was slightly higher than the RACC value (36 g vs. 30 g).

Mean serving sizes for most mixed foods, as observed in the NHANES data, were consistently above FDA RACC values. There were some major differences, for example for pasta and macaroni and cheese where the observed amounts consumed were far in excess of RACC values. In some cases, the differences were only minor (e.g., sandwiches) and there were a few cases where the RACC value actually exceeded the amount consumed (seafood and pulse-based dishes and vegetable sandwiches).

### 3.2. NDC643 and NDC343 Alternative Models

**Figure 1:**
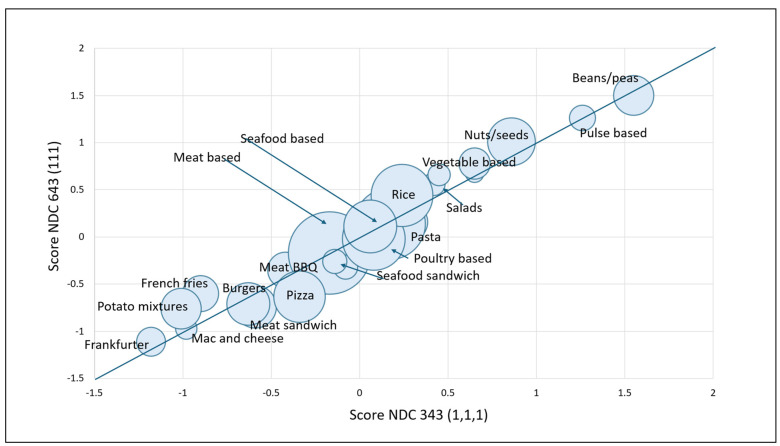
A scatterplot of mean NDC343 scores against mean NDC643 scores by category. (1,1,1) shows an equivalent weight between the three components of the Nutrient Density Calculator. The size of the bubble denotes the number of items per category. Spearman correlation was 0.97.

Figure 1 shows a scatterplot of NDC343 scores (X-axis) against NDC643 scores (Y-axis) by category. Both scores use the default (1,1,1) weighting. Differences between the two alternative scores are indicated by food categories that fall above or below the diagonal. The size of the bubble denotes the number of items in each category. As seen, the highest NDC scores were obtained for plant-based mixed foods containing pulses, vegetables and nuts, and with few limiting nutrients. Lowest scores were given to foods that were high in sodium and in saturated fats, notably frankfurters, mac and cheese, French fries, processed meat sandwiches and burgers. Potato mixtures (mashed potatoes with butter) also scored low. Mixed dishes with qualifying nutrients such as protein (meat-based mixed dishes) received middle range scores as did non-lettuce salads (coleslaw).

The two models produced almost identical scores: the Spearman correlation coefficient was 0.97. The inclusion of iron, potassium, and vitamin D in the NDC643 model led to higher ratings for some meat dishes (iron), potatoes, nuts, and seeds (potassium).

### 3.3. Different Weights for NDC343 Components (1,1,1), (1,1,2), (2,1,1) and (1,2,1)

**Figure 2:**
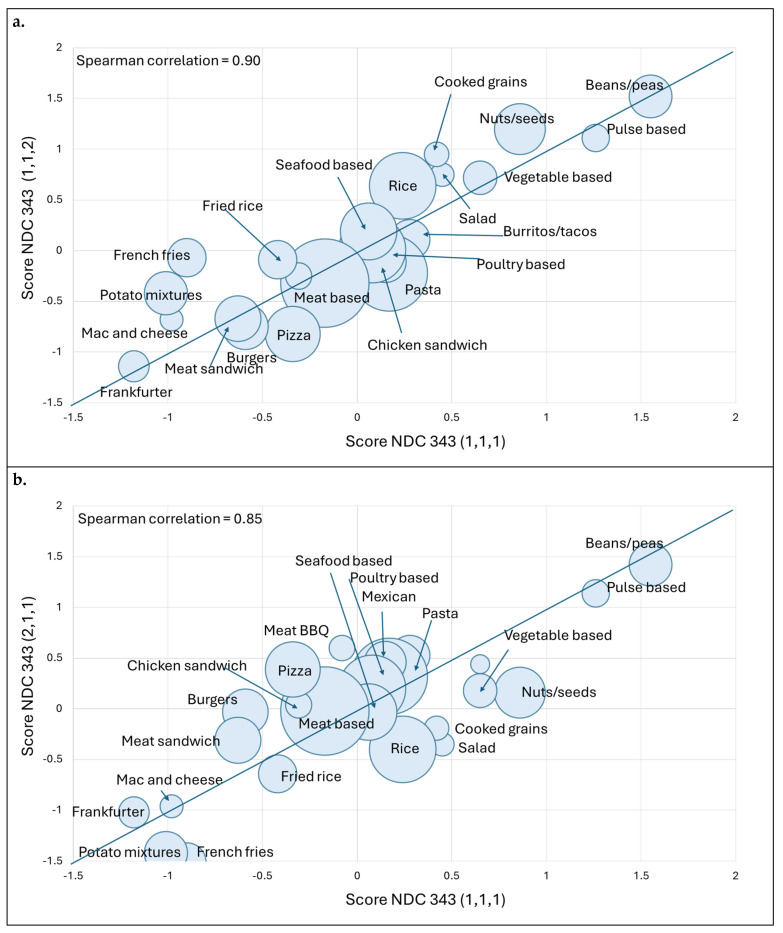

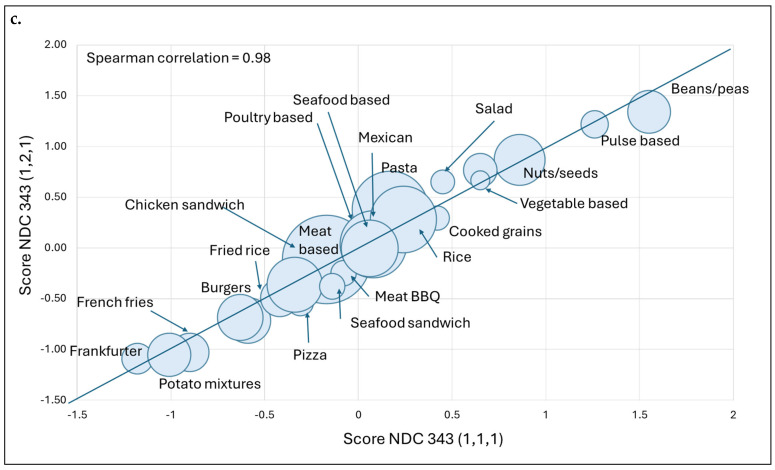
A scatterplot of mean NDC343 (1,1,1) scores against mean NDC343 scores with different weights: 1,1,2 (**a**), 2,1,1 (**b**) and 1,2,1 (**c**) by category. (1,1,1) shows an equivalent weight between the three components of the Nutrient Density Calculator. The size of the bubble denotes the number of items per category. All scores are standardized (i.e., centered and reduced) to make the comparison possible.

Different weighting schemes applied to the NDC343 model are shown in Figure 2. Figure 2a shows the default scheme (1,1,1) on the X-axis and (1,1,2) on the Y-axis. Weighting the negative LIM component reduced total scores for meats (BBQ, frankfurters, cured meats, burgers) and for burritos, pizza, Mexican, and pasta-based dishes. Conversely, scores increased for rice- and seafood-based dishes, French fries, fried rice, potato mixtures, cooked grains, macaroni and cheese, non-lettuce salads, and for nuts and seeds.

Figure 2b plots the default scheme (1,1,1) against (2,1,1) on the Y-axis. Weighting the NR3 component led to higher scores for meat-based dishes, including burgers, meat sandwiches, BBQ sandwiches, chicken sandwiches and pizza. All these were now above the diagonal. By contrast, lower scores were now given to rice and to nuts and seeds (below the diagonal).

Figure 2c plots the default scheme (1,1,1) against (1,2,1). Weighting the MP4 component had little impact on the final score. All foods were very close to the diagonal and no major trends were observed (Spearman correlation = 0.98).

### 3.4. Different Weights for NDC643 Components (1,1,1), (1,1,2), (2,1,1) and (1,2,1)

**Figure 3:**
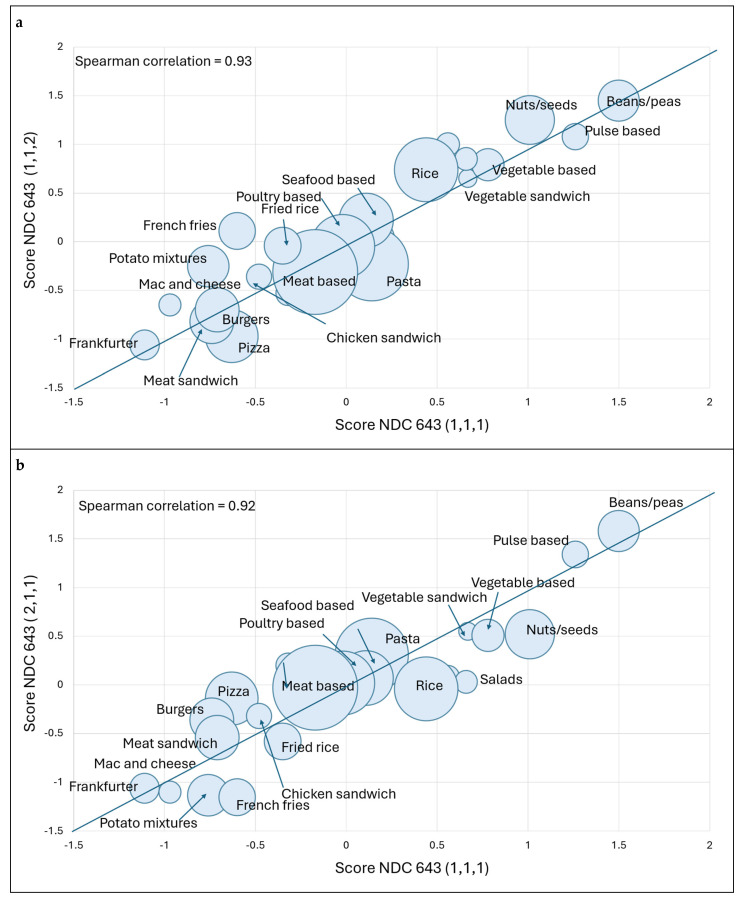

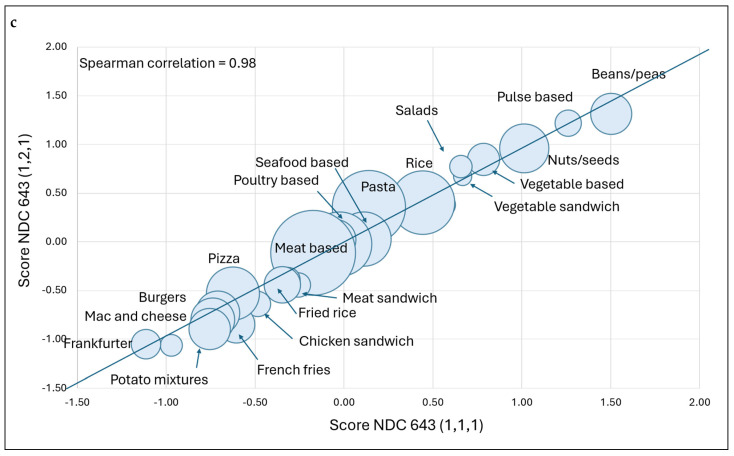
A scatterplot of mean NDC643 (1,1,1) scores against mean NDC643 scores with different weights: 1,1,2 (**a**), 2,1,1 (**b**) and 1,2,1 (**c**) by category. (1,1,1) shows an equivalent weight between the three components of the Nutrient Density Calculator. The size of the bubble denotes the number of items per category. All scores are standardized (i.e., centered and reduced) to make the comparison possible.

Figure 3 shows different weighting schemes applied to the NDC643 model. Figure 3a shows the default scheme (1,1,1) on the X-axis and (1,1,2) on the Y-axis. Weighting the negative LIM component reduced total scores for meats (BBQ, frankfurters, cured meats, burgers) and for burritos, pizza, and Mexican, and pasta-based dishes. Conversely, the (1,1,2) version favored plant-based foods, with less fat, sugar and salt. NDC643 (1,1,2) were selectively higher for rice- and seafood-based dishes, French fries, fried rice, potato mixtures, cooked grains, macaroni and cheese, non-lettuce salads, and for nuts and seeds. Poultry, pulses, vegetable-based dishes, chicken and seafood sandwiches all maintained similar mid-range rankings (±1 position) regardless of the weighting scheme.

Figure 3b plots the default scheme (1,1,1) against (2,1,1) on the Y-axis. Weighting the NR6 component led to higher scores for meat-based dishes, including burgers, meat sandwiches, BBQ sandwiches, chicken sandwiches and pizza. All these were now above the diagonal. By contrast, lower scores were now given to rice and to nuts and seeds (all below the diagonal). Figure 2c plots the default scheme (1,1,1) against (1,2,1). This version maximizes the MP4 component. All foods were very close to the diagonal and no major trends were observed (Spearman correlation = 0.98).

### 3.5. Contrast Between NDC643 (1,1,2) and NDC643 (2,1,1) Alternative Weights

**Figure 4:**
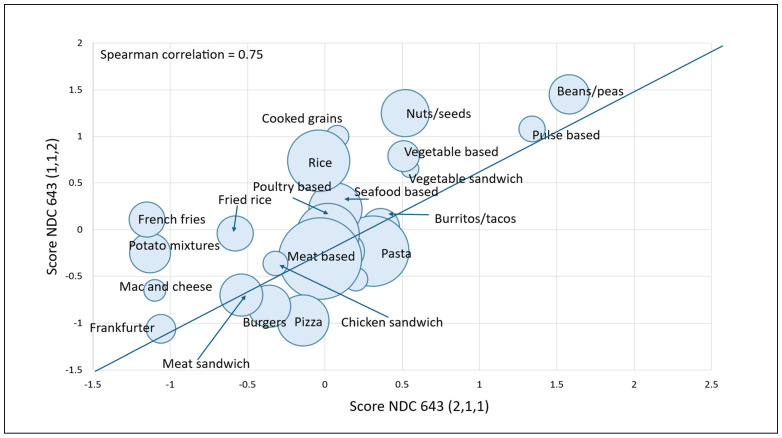
A scatterplot of mean NDC643 (1,1,2) scores against mean NDC643 (2,1,1,) scores to demonstrate maximum contrast. (1,1,2) and (2,1,1) show the different sets of weights used for the three components of the Nutrient Density Calculator. The size of the bubble denotes the number of items per category. All scores are standardized (i.e., centered and reduced) to make the comparison possible.

The contrast between alternative NDC643 weighting schemes is brought out in Figure 4. The NDC643 (1,1,2) version penalizes saturated fat, added sugars and sodium whereas the (2,1,1) version gives more weight to fiber, protein and micronutrients. Found above the diagonal are nuts, vegetable dishes, rice and grain dishes. Found below the diagonal are burgers, meat dishes and pizza.

### 3.6. Correlations Between NDC643 and Nutri-Score and Health Star Rating (HSR)

Table 5 shows how NDC643 total score and components (NR6, MP4, and LIM3) were correlated with Nutri-Score and HSR values. First, both Nutri-Score and HSR penalize saturated fat, sugar and sodium, and so does the LIM3 component of the present NDC. As a result, both Nutri-Score and HSR were highly correlated with LIM3 (0.77 and 0.69, respectively). Weighting the LIM3 component more (1,1,2 models) led to higher correlations with both Nutri-Score and HSR. The correlations were negative, given that both Nutri-Score use higher point values to identify less healthy foods.

Both Nutri-Score and HSR feature vegetables and fruit (but not whole grains) as potential positive components. The correlation with MP4 was moderate (−0.41, −0.47 respectively). Other than protein and fiber, Nutri-Score and HSR do not include beneficial nutrients, vitamins, or minerals. The correlations with NR6 were therefore lower (0.33 and 0.16, respectively).

Both NDC643 and NDC343 total scores were significantly correlated with Nutri-Score and HSR. The correlations for the NDC643 default unweighted score (1,1,1) were −0.70 for Nutri-Score and −0.75 for HSR. The correlations were highest (−0.79) for those NDC models (1,1,2) that weighted LIM3 more strongly. In other words, nutrient density was driven by saturated fat, sugar and salt. The correlations were lowest (−0.39) for those NDC models (2,1,1) that assigned higher weights to the NR component featuring vitamins and minerals. That would suggest that Nutri-Score and HSR track problematic nutrients but fail to capture the full nutrient density of foods.

As expected, both Nutri-Score and HSR were positively correlated with energy density (0.55 and 0.36 respectively). The correlations between NDC643 and energy density were lower, ranging from −0.16 (2,1,1) to –0.42 (1,1,2). It is important to ensure that any NP model is not simply a function of energy density. The NDC643 (1,1,1) and (1,1,2) models had the highest correlations with Nutri-Score and HSR, and low correlations with energy density. These were selected as the preferred versions.

### 3.7. Converting Raw NDC Values to NDC643 10-Point Score

The conversion was based on the distribution of NDC643 scores for the 1570 items. The distributions of the NDC643 scores were close to a normal distribution (Supplemental Figure S1). The means and ranges differed depending on the weighting scheme. The NDC643(1,1,1) ranged from –75 to 50, with a mean of 6.9. The versions of the NDC that assigned more weight to NR6 reached up to 100, whereas those giving more weight to LIM3 started at –100. Starting from the deciles for each version of NDC score (Table 6), theoretical thresholds were derived to translate NDC scores into 10-point grades. These thresholds are in Table 7 and are graphically represented in Supplemental Figure S2.

### 3.8. Sensitivity of NDC643 Within Food Categories Using 10-Point Scale

**Figure 5:**
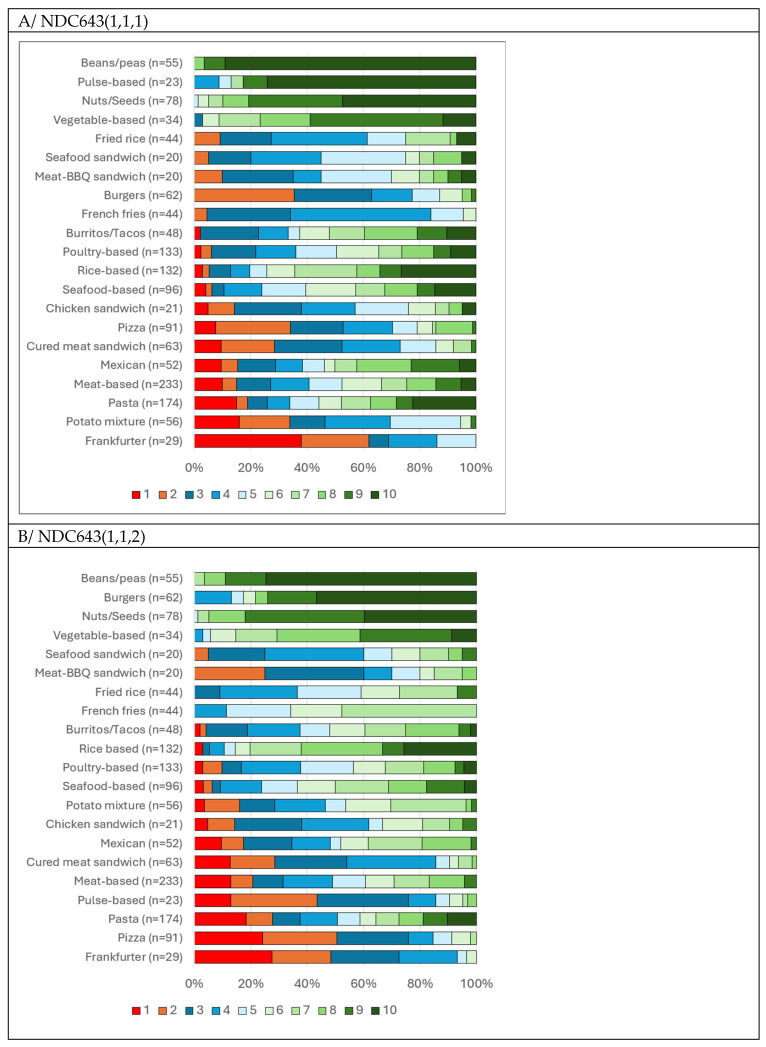

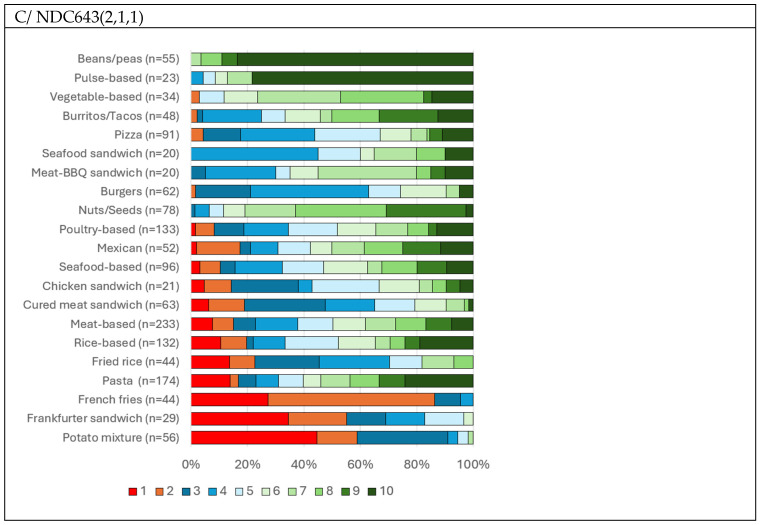
Percentage of foods (*N* = 1570) scoring 1–10 points by category and score type. NDC643(1,1,1) (**A**), NDC643(1,1,2) (**B**), NDC643(2,1,1) (**C**).

Figure 5 shows the distribution of foods across the 10 NDC grades by food category. With some exceptions, the NDC assigned foods in each category to at least five different grades. NDC grades ranged from 1 to 10 for eight food categories, mainly multi-ingredient mixed dishes and sandwiches. The NDC643(1,1,1) version led to similar rankings overall with NDC643(1,1,2) (weighted Kappa = 0.76) and NDC643(2,1,1) (weighted Kappa = 0.73); however, NDC643(1,1,2) and NDC643(2,1,1) were less concordant (weighted Kappa = 0.52). NDC643(1,1,2) gave lower ratings to pizza, burgers, and pasta-based dishes and higher ratings to potato mixtures and cured meats compared with NDC643(1,1,1).

### 3.9. Sensitivity of NDC643 to Reformulation of Mixed Dishes

These analyses tested original and reformulated recipes supplied by Griffith Foods. The NDC643 models were more sensitive to the reformulation of recipes than either Nutri-Score or HSR (Table 8). NDC643 scores changed by as much as 6 points following the reformulation of spiced chickpea and lentil stew and improved by 1 or 2 points for cheese-flavored rice. Nutri-Score was largely insensitive to reformulation and HSR scores hardly moved.

## 4. Discussion

The present NDC was intended to assist the hospitality and prepared meals industries in reformulating recipes to improve the nutrient density of the final product. Several such initiatives are currently underway [27,28,29,30,31]. The NDC was designed to be sensitive to nutrient density and to rebalancing of recipe ingredients.

Modification of prepared meals can take several forms. Changing the ratio of meat, eggs, or dairy to plant proteins and/or including more whole grains, beans, or vegetables will change the nutrient composition of the reformulated product [32,33,34]. In general, animal source foods contain more protein, iron, calcium and vitamin D, whereas plant-based foods are likely to be higher in potassium and fiber [35]. However, animal food sources tend to be higher in saturated fat and can be higher in sodium. For that reason, a Nutrient Density Calculator for mixed dishes ought to be sensitive to both nutrients and ingredients.

The present Nutrient Density Calculator (NDC), specifically designed for the purpose, is a three-component score that incorporates nutrients to encourage (NRn component), ingredients to encourage (MP4 component), and nutrients to limit (LIM component). The goal was to make the NDC methodologically rigorous yet flexible enough to capture product innovation strategies that are consistent with the current dietary guidance.

Differential weightings of the three components were designed to accommodate evolving dietary guidelines. For example, the 2025-30 DGA advisory committee favored plant-based ingredients. The recommendation was to place pulses ahead of other protein sources, including red meat, eggs, and dairy [24]. By contrast, the newly released DGA 2025-30 place more emphasis on red meat and full-fat dairy [36,37]. Analyses of cross-sectional NHANES data have explored the consumption of beef [38,39], dairy [40] and beans [41] in relation to dietary nutrient adequacy and diet quality.

Across all NDC versions, plant-based mixed dishes consistently achieved higher scores, whereas meat-based dishes generally ranked lower. However, the (2,1,1) weighting that emphasized protein and iron gave higher scores to meat-based dishes, including burgers, meat and BBQ sandwiches, and pizza.

Four different weighting options for NDC343 and NDC643 models were tested, shifting emphasis between nutrients to encourage (NRn component), desirable ingredients (MP4 component), and nutrients to limit (LIM component). The default NDC(1,1,1), which assigned equal weights to the three components, provided a balanced distribution of scores, supporting its use as a general benchmarking tool. The NDC(1,1,2), which weighted the negative LIM component more, gave lower scores to meat dishes but was more sensitive to recipes reduced in fat, sugar, and salt. In contrast, NDC(2,1,1) gave more weight to nutrients to encourage favored meat-based dishes with more protein and iron. The NDC(1,2,1) emphasized ingredients favored plant-based dishes but has little impact on final scores. One caution is that the NDC(1,2,1) version may be less sensitive to the removal of fat, sugar or salt (see flavored peanuts).

Capturing nutrient density is a process that involves numerous decisions. First, we decided to calculate nutrient density per serving and not per 100 g. The rationale was that a servings-based approach was more aligned with how consumers eat restaurant foods away from home. Observed serving sizes (rather than RACC values) were the base of calculations [42]. One concern was that the RACC values had not been updated for some time and were no longer reflecting the current eating habits of US adults [43]. Indeed, while the amounts consumed for some food categories closely matched RACC values, substantial discrepancies were also observed. It may be necessary to conduct a future study to compare FDA RACC values, industry portion sizes, and amounts actually consumed; the three sets of serving sizes may not be the same.

There was another reason to use servings as the reference amount. Should the FDA develop a nutrient profiling system for prepared mixed dishes, it would likely rely on RACC values or on other standardized serving sizes. Anticipating such regulatory developments, observed serving sizes were compared to RACC values. By working in advance of potential regulatory action, the NDC provides a practical, data-driven framework for profiling mixed dishes as they are consumed.

The second important decision was to include both nutrients and ingredients. Our compensatory approach balanced nutrients to encourage (NRn component) against nutrients to limit (LIM component). Many NP models view nutrient density as the absence of problematic nutrients, awarding higher scores to foods that are low in calories, total sugar, saturated fat and sodium. As a result, both Nutri-Score and HSR are highly correlated with energy density, while failing to capture the nutritional value of foods. The NDC did show moderate correlations with Nutri-Score and the Health Star Rating (HSR), but it was not colinear with either. As such the NDC was not simply a transformation of energy density. Rather, NDC scores clearly captured gradients of nutrient density across the analytical database of 1570 foods. Some items in the same category received markedly higher scores than others, indicating sensitivity to recipe composition.

Third, the MP4 component helped to distinguish the NDC from nutrient-only NP models. This was done to better track reformulation by explicitly giving higher scores to mixed dishes containing desirable ingredients. The NDC seemed to capture recipe renovation within a given product category more accurately than did Nutri-Score and HSR. Improved recipes—whether achieved through sodium reduction, increased fiber, or the addition of nutrient-dense ingredients—were rewarded with higher point scores. The same improvements were not captured by Nutri-Score.

The study had several methodological limitations. Converting the FPED ingredient measures from ounce- or cup-equivalents into grams was made complicated by food-specific conversion factors. This remains a general challenge in ingredient-based profiling, where recipe data are expressed in grams. Raw NDC scores were converted to a 10-point scale using decile splits derived from the current database. While this approach is practical, it introduces a limitation, since the cut points may shift with different datasets. Finally, the evaluation of NDC responsiveness to food reformulation was conducted on a limited number of products and should therefore be considered as a proof-of-concept analysis. Further studies conducted on larger and representative samples of reformulated foods are needed to assess whether these findings can be generalized.

The present intent was to help the hospitality industry implement the Dietary Guidelines for Americans in a practical manner. Foods purchased and eaten away from home account for an increasing proportion of daily energy intakes. The present scoring system can help health professionals and the public to evaluate the nutrient density of prepared meals offered away from home.

## 5. Conclusions

Providing more opportunities for reformulating foods eaten away from home can help improve the overall nutritional quality of the food supply. The proposed NDC can serve to benchmark improvements in nutrient content per serving. Food industry actors have a pivotal role in fostering healthier population diets. In this context, the development of food scoring systems represents a key public health strategy to promote adherence to dietary guidelines and, ultimately, improve population health.

## Figures and Tables

**Table 1:** Food subgroups and categories, 4-digit WWEIA codes [6], and the number of items per category.

Food Group	4-Digit WWEIA Code	Shortened Name	WWEIA Category Full Name	*N*
Mixed dishes	3006	Seafood-based	Seafood mixed dishes	96
Mixed dishes	3202	Rice	Rice mixed dishes	132
Mixed dishes	3204	Pasta	Pasta mixed dishes, excl mac and cheese	174
Mixed dishes	3206	Mac and cheese	Macaroni and cheese	16
Mixed dishes	3104	Vegetable-based	Vegetable dishes	34
Mixed dishes	4004	Cooked grains	Pasta, noodles, cooked grains	18
Mixed dishes	3402	Fried rice	Fried rice and lo/chow mein	44
Mixed dishes	3002	Meat-based	Meat mixed dishes	233
Mixed dishes	3004	Poultry-based	Poultry mixed dishes	133
Mixed dishes	3102	Pulse-dbased	Bean, pea, legume dishes	23
Mixed dishes	3502	Burritos/tacos	Burritos and tacos	48
Mixed dishes	3506	Mexican	Other Mexican mixed dishes	52
Mixed dishes	3602	Pizza	Pizza	91
Sandwiches	3702	Burgers	Burgers	62
Sandwiches	3742	Meat–BBQ sandwich	Meat and BBQ sandwiches	20
Sandwiches	3740	Cured meat sandwich	Deli and cured meat sandwiches	63
Sandwiches	3704	Chicken sandwich	Chicken filet sandwiches	21
Sandwiches	3730	Seafood sandwich	Seafood sandwiches	20
Sandwiches	3703	Frankfurter	Frankfurter sandwiches	29
Sandwiches	3744	Vegetable sandwich	Vegetable sandwiches/burgers	11
Single dishes	2802	Beans/peas	Beans, peas, legumes (cooked)	55
Single dishes	6432	Non-lettuce salads	Coleslaw, non-lettuce salads	17
Single dishes	6806	Potato mixtures	Mashed potatoes, white potato mixtures	56
Single dishes	6804	French fries	French fries, fried white potatoes	44
Nuts and seeds	2804	Nuts/seeds	Nuts and seeds	78

**Table 2:** The three score components and reference values [22] used in the Nutrient Density Calculator. References values are needed to reach the maximum values of the NRx, MP4 and LIM3 component.

Nutrients to Encourage	Daily Value	Ingredients	Daily Value	Nutrients to Limit	Maximum Recommended Value
Protein *	50 g	Fruits and vegetable	85 g	Saturated fats	20 g
Fiber *	28 g	Whole grains	50 g	Sodium	2300 mg
Calcium *	1300 mg	Pulses	65 g	Total/added sugars	Total 90 g/added 50 g
Iron	18 mg	Nuts and seeds	30 g		
Potassium	3500 mg				
Vitamin D	20 mcg				

* Nutrients mandatory in the calculation of the NRx.

**Table 3:** Different sets of weights used in the calculation of the NDC643 and NDC343.

Name	Formula	Weight for NRn3 or 6	Weight for MP4	Weight for LIM3	Range for Final Score, %
NDCn43(1,1,1)	NRn×1 + MP4×1 − LIM3×1	1/3 = 0.33	1/3 = 0.33	1/3 = 0.33	−100 to 200
NDCn43(1,1,2)	NRn×1 + MP4×1 − LIM3×2	1/4 = 0.25	1/4 = 0.25	2/4 = 0.50	−200 to 200
NDCn43(2,1,1)	NRn×2 + MP4×1 − LIM3×1	2/4 = 0.50	1/4 = 0.25	1/4 = 0.25	−100 to 300
NDCn43(1,2,1)	NRn×1 + MP4×2 − LIM3×1	1/4 = 0.25	2/4 = 0.50	1/4 = 0.25	−100 to 300

**Table 4:** Observed portion sizes (in grams) as consumed by adults in NHANES 2017–2023 by food category compared to FDA Reference Amounts Customarily Consumed (RACCs). Shown are means, SEM, confidence intervals and 10th, 50th and 90th percentiles. The number of observed events used as the base of calculation is indicated as well.

	RACC	Observed Serving Sizes from NHANES 2017–2023 for Adults							
Category		*N* Events	Mean	SEM	LCL	UCL	10th ile	50th ile	90th ile
Seafood-based	240	523	211	14.67	187	236	42	170	442
Rice-based	240	1163	181	6.04	171	191	56	157	344
Pasta	140	2476	323	6.30	313	334	102	250	500
Mac and cheese	140	816	234	10.63	216	252	77	201	460
Vegetable-based	113	384	187	14.03	164	211	54	149	359
Cooked grains	140	546	153	11.05	134	171	43	120	280
Fried rice	195	764	236	9.80	220	253	74	198	433
Meat-based	240	1935	255	7.64	242	268	60	202	504
Poultry-based	240	1452	246	7.11	234	258	75	210	460
Pulse-based	240	590	205	14.88	180	230	64	170	340
Burritos/tacos	140	1102	193	7.59	180	206	70	160	362
Mexican	170	1004	199	7.64	186	212	63	157	384
Pizza	195	2546	212	4.68	204	220	70	175	402
Burgers	170	2047	178	2.67	173	182	101	165	270
Meat–BBQ sandwich	140	399	216	5.96	206	226	100	180	360
Cured meat sandwich	155	2166	156	3.05	150	161	86	140	250
Chicken sandwich	160	892	161	3.97	154	168	92	140	280
Seafood sandwich	140	498	185	5.88	175	195	120	180	302
Frankfurter	140	697	162	4.65	154	170	88	120	302
Vegetable sandwich	155	138	146	6.28	136	157	64	140	250
Beans/peas	130	1394	135	5.04	127	144	25	104	260
Non-lettuce salads	100	685	122	7.40	109	134	28	110	220
Potato mixtures	140	1759	191	4.33	184	198	63	188	340
French fries	70	3660	95	1.93	91	98	30	80	160
Nuts/seeds	30	5785	36	1.19	34	38	5	28	73

RACC Reference Amount Customarily Consumed; SEM standard error of the mean; LCL lower confidence limit; UCL upper confidence limit. Ile percentiles.

**Table 5:** Spearman correlations between NDC343 and NDC643 total scores and sub-scores component and Nutri-Score and HSR (*N* = 1570). All correlations are significant except when “NS” is specified.

Scores and Metrics	Energy Density, kcal/100 g	Energy Density, kcal/Serving	Nutri-Score *	HSR Score
NR6	0.44	0.73	0.33	0.16
MP4	−0.23	−0.31	−0.41	−0.47
LIM3	0.46	0.85	0.77	0.69
NDC343 (1,1,1)	−0.25	−0.35	−0.64	−0.74
NDC343 (1,1,2)	−0.38	−0.64	−0.78	−0.80
NDC343 (2,1,1)	0.00 (NS)	0.07	−0.39	−0.58
NDC343 (1,2,1)	−0.25	−0.31	−0.56	−0.66
NDC643 (1,1,1)	−0.33	−0.53	−0.70	−0.75
NDC643 (1,1,2)	−0.42	−0.71	−0.79	−0.79
NDC643 (2,1,1)	−0.16	−0.26	−0.57	−0.70
NDC643 (1,2,1)	−0.30	−0.40	−0.60	−0.67
ED kcal/100 g	1.00	0.61	0.55	0.36
ED kcal/serving	0.61	1.00	0.63	0.54

* Continuous score of Nutri-Score.

**Table 6:** Values of deciles of NDC643 used as thresholds to translate NDC scores into a 10-point scale.

NDC Version	1st	2nd	3rd	4th	5th	6th	7th	8th	9th
NDC643(1,1,1)	−12.54	−7.75	−3.50	0.13	4.83	10.58	16.75	22.50	29.25
NDC643(1,1,2)	−47.92	−36.00	−27.63	−20.88	−14.96	−8.75	−0.50	8.42	20.46
NDC643(2,1,1)	1.33	7.29	11.33	15.25	19.67	24.42	29.83	35.50	43.00
NDC643(1,2,1)	−10.50	−5.83	−0.58	7.33	17.50	28.42	39.33	47.50	55.25

**Table 7:** Thresholds used to translate the NDC raw score into a 10-point scale. Thresholds were derived by authors from decile values presented in Table 6.

NDC Grade	1	2	3	4	5	6	7	8	9	10
NDC643(1,1,1)	<−15	<−10	<−5	<0	<5	<10	<15	<20	<25	≥25
NDC643(1,1,2)	<−50	<−40	<−30	<−20	<−15	<−10	<0	<10	<20	≥20
NDC643(2,1,1)	<0	<5	<10	<15	<20	<25	<30	<35	<40	≥40
NDC643(1,2,1)	<−10	<−5	<0	<5	<15	<25	<35	<45	<55	≥55

**Table 8:** NDC scores and grades of original and revised recipes for selected dishes.

Food Description	Version	Serving (g)	Nutri-Score	HSR Star	NDC643(2,1,1)		NDC643(1,2,1)		NDC643(1,1,1)		NDC643(1,1,2)	
					Score	Grade	Score	Grade	Score	Grade	Score	Grade
Spiced chickpea and lentil	Original	284	C	6	28.3	7	51.0	9	11.8	7	−32.3	3
	Modified	291	B	7	50.3	10	73.2	10	33.9	10	12.3	9
Flavored rice A	Original	160	C	6	0.7	2	−7.2	2	−7.2	3	−22.2	4
	Modified	156	C	7	2.0	2	−4.0	3	−4.0	4	−14.0	6
Flavored rice B	Original	160	C	6	6.7	3	−7.2	2	2.5	5	−6.2	7
	Modified	156	C	7	10.0	4	−3.3	3	5.8	6	0.5	8
Flavored rice C	Original	151	C	6	0.7	2	−8.7	2	−7.2	3	−22.2	4
	Modified	144	C	7	2.7	2	−0.8	3	−3.3	4	−12.7	6
Flavored meatballs	Original	140	D	6	−11.0	1.0	−16.7	1.0	−16.7	1.0	−39.0	3.0
	Modified 1	140	D	7	−7.0	1.0	−13.5	1.0	−13.5	2.0	−33.5	3.0
	Modified 2	140	D	7	−6.7	1.0	−13.3	1.0	−13.3	2.0	−33.3	3.0
Flavored peanuts	Original	30	D	3	6.7	3.0	9.5	5.0	2.5	5.0	−6.2	7.0
	Modified	30	C	5	10.0	4.0	12.8	5.0	5.8	6.0	0.5	8.0

## Data Availability

Publicly available datasets can be found at https://www.ars.usda.gov/northeast-area/beltsville-md-bhnrc/beltsville-human-nutrition-research-center/food-surveys-research-group/docs/fndds-download-databases/ (accessed on 1 February 2025).

## Supplementary Materials

The following supporting information can be downloaded at: https://www.mdpi.com/article/10.3390/nu18142282/s1, Supplemental Table S1: Nutrient changes per serving expressed in percentage of the initial nutritional composition of recipes provided by Griffith Foods. Supplemental Figure S1: Distribution of the family of NDC scores. Red line shows the mean. Supplemental Figure S2: Graphical representation of decile values derived from the distribution of the different versions of NCD 643 estimated among FNDDS mixed dishes (N = 1540).

## Abbreviations

The following abbreviations are used in this manuscript:

DVs	Daily values
DGA	Dietary guidelines for Americans
FDA	Food and Drug Administration
FNDDS	Food and Nutrient Database for Dietary Studies
FPED	Food Patterns Equivalents Database
MRVs	Maximal recommended values
NP	Nutrient profile
NDC	Nutrient Density Calculator
NHANES	National Health and Nutrition Examination Survey
RACC	Reference Amount Customarily Consumed
