# Supplementary material for: Development of a Three-Component Nutrient Density Calculator (NDC) for Mixed Dishes to Guide Innovation and Reformulation of Recipes
Source: Nutrients. 2026 Jul 12;18(14):2282. doi: 10.3390/nu18142282 (PMC13416164; doi:10.3390/nu18142282)
Supplement: Supplementary file 1 [file nutrients-18-02282-s001.zip › nutrients-4370705-supplementary.pdf]

SUPPLEMENTAL DATA

Supplemental Table S1. Nutrient changes per serving expressed in percentage of the initial nutritional composition of recipes provided by Griffith Foods

Nutrient/ingredients	Flavored Rice A	Flavored Rice B	Flavored Rice C	Spiced Chickpea & Lentil	Flavored Peanuts	Flavored Meatballs (Modified 1)	Flavored Meatballs (Modified 2)
**Protein**	↓17%	↓17%	↓30%	↑14%			
**Fiber**	↓75%	↓75%	↓75%			↑136% (1.5g)	↑145% (1.6)
**Saturated Fat**				↓77%			
**Sodium**	↓41%	↓41%	↓63%	↑5%	↓70%	↓27% (137 mg)	↓27% (137 mg)
**Total Sugar**						↓14% (1g)	↓18% (1.3g)
**Added Sugar**							
**Calcium**	↓54%	↓54%	↓49%	↑95%			
**Vitamin D**				↑122%			
**Iron**	↓41%	↓41%		↓56%			
**Potassium**	↑209%	↑209%	↓18%	↓10%			
**Whole Grain**							
**Pulses**							
**Fruit/Veg**							
**Nuts/Seeds**							

Supplemental Figure S1. Distribution of the family of Nutrient Density Calculator scores. Red line shows the mean.

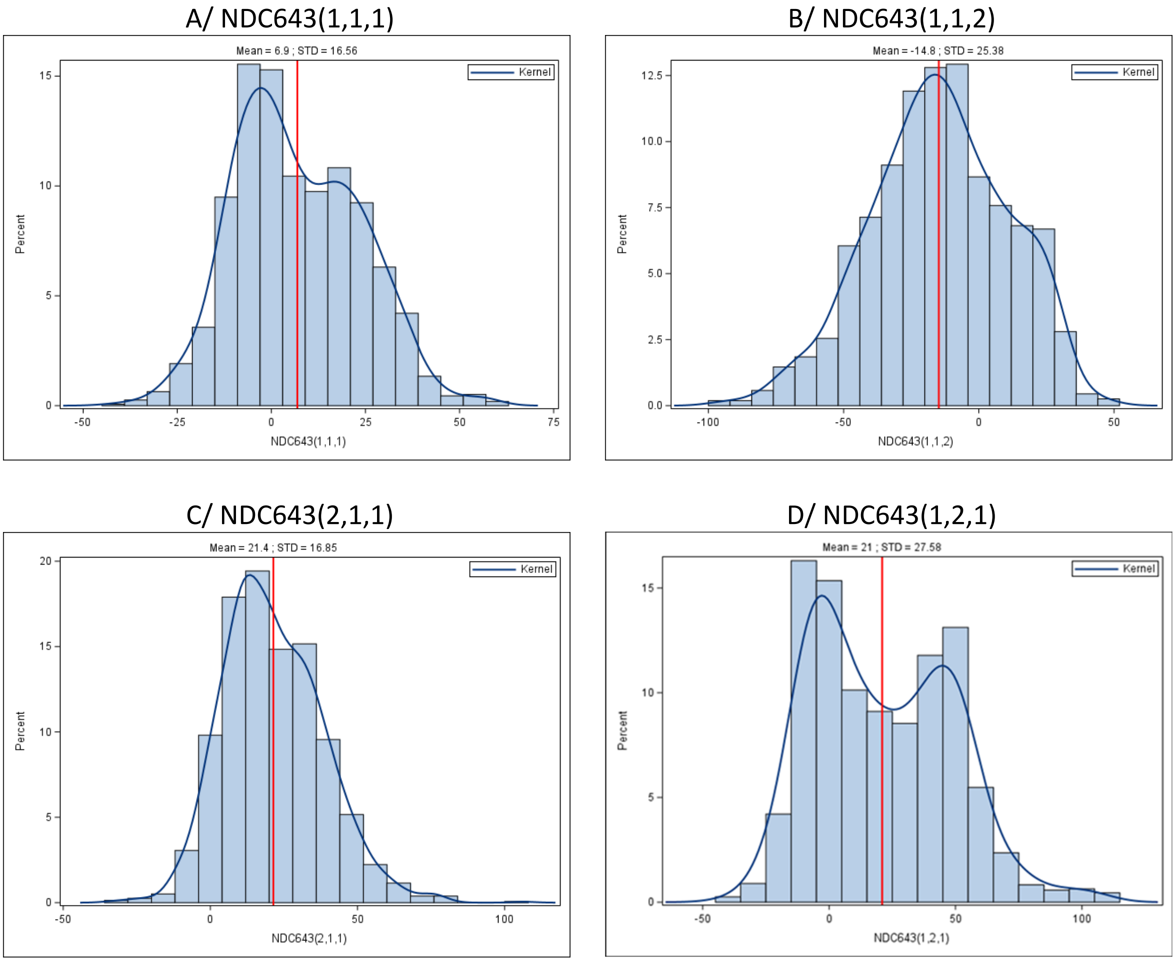

The distributions of the NDC643 scores were close to a normal distribution. The means and ranges differed depending on the weighting scheme. The NDC643(1,1,1) ranged from –50 to 75, with a mean of 6.9. The versions of the NDC that assigned more weight to NR6 reached up to 100, whereas those giving more weight to LIM3 started at –100. The NDC643(1,2,1) ranged from –50 to 100. The standard deviation was similar for NDC643(2,1,1) and NDC643(1,1,1) (around 16), but slightly higher for the other versions (27 for NDC643(1,2,1) and 25 for NDC643(1,1,2)).

Spearman correlation coefficients comparing NDC final scores based on 6 versus 3 qualifying nutrients ranged from 0.92 to 0.99 across the different weighting schemes. The lowest correlation was obtained for the score with the weight being the highest for NR component. Spearman correlation between NR3 and NR6 was 0.98. These results confirm that the NDC can be computed using different numbers of qualifying nutrients without substantially altering the ranking. All next results are presented using the NDC based on 6 qualifying nutrients.

Supplemental Figure S2. Graphical representation of decile values derived from the distribution of the different versions of NCD 643 estimated among FNDDS mixed dishes (N=1,540)

a.NDC643(1,1,1)

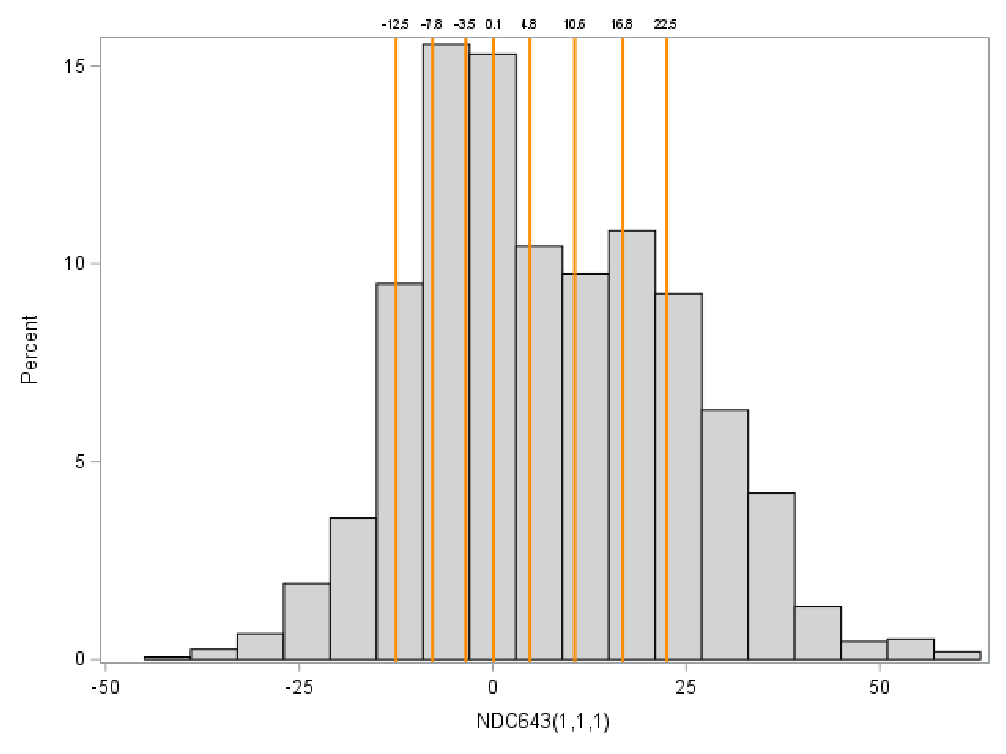

b. NDC643(1,1,2)

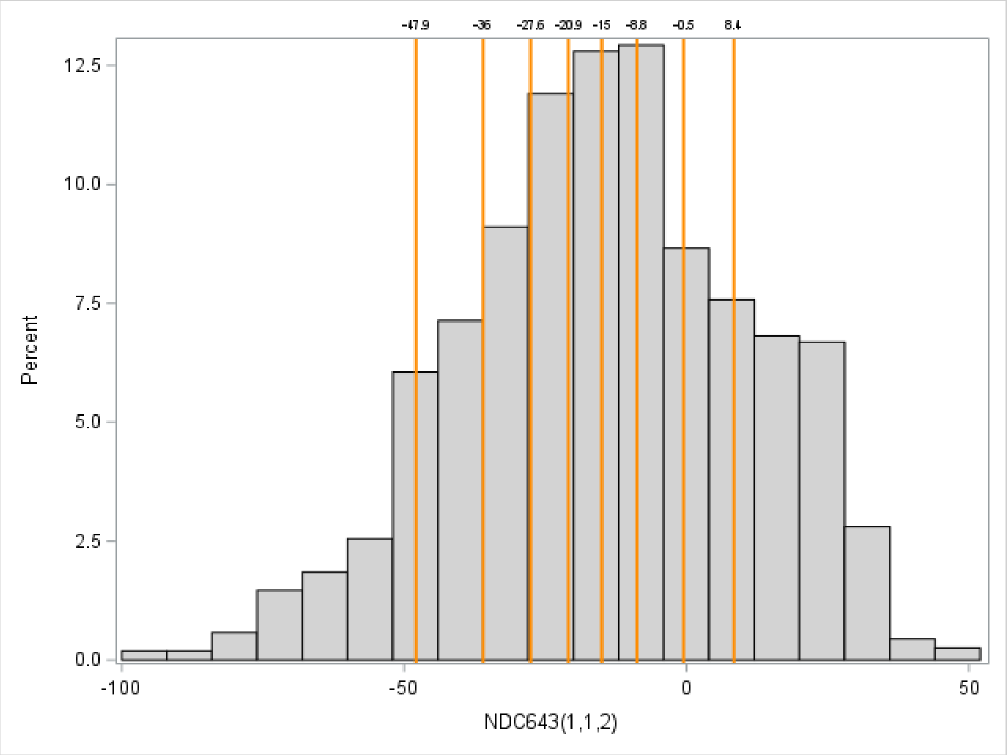

c. NDC643(2,1,1)

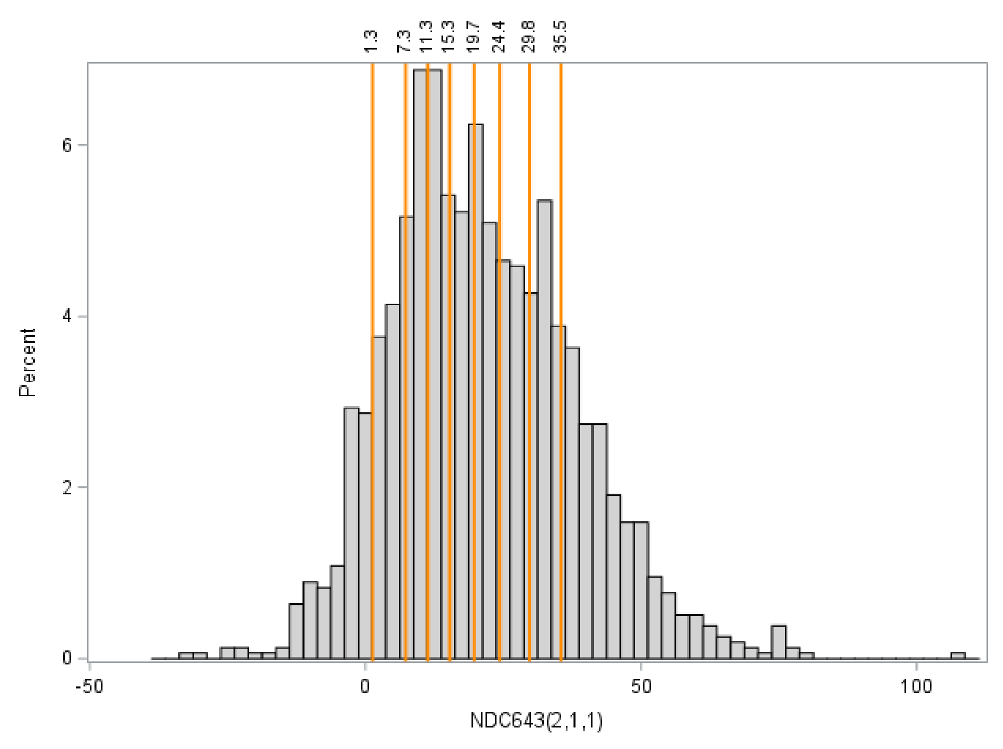
1.3
7.3
11.3
15.3
19.7
24.4
29.8
35.5
Percent
6
4
2
0
-50
0
50
100
NDC643(2,1,1)

d. NDC643(1,2,1)

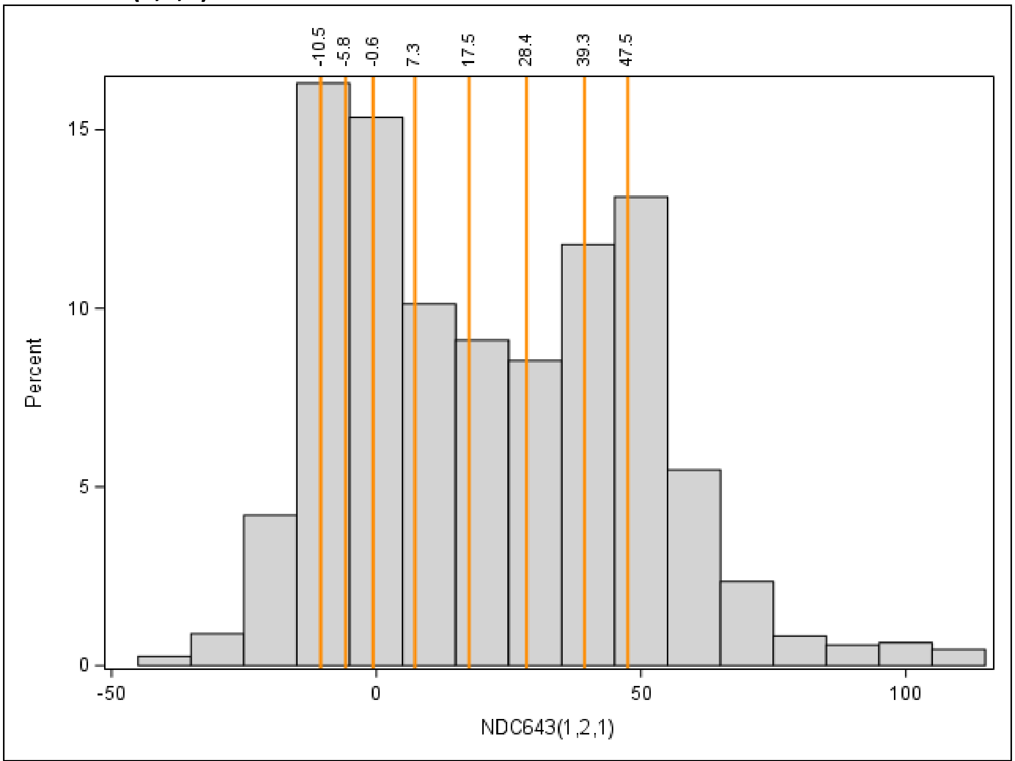
-10.5
-5.8
-0.6
7.3
17.5
28.4
39.3
47.5
Percent
15
10
5
0
-50
0
50
100
NDC643(1,2,1)
